# Gamification in the Design of Virtual Patients for Swedish Military Medics to Support Trauma Training: Interaction Analysis and Semistructured Interview Study

**DOI:** 10.2196/63390

**Published:** 2024-10-22

**Authors:** Natalia Stathakarou, Andrzej A Kononowicz, Erik Mattsson, Klas Karlgren

**Affiliations:** 1 Department of Learning, Informatics, Management and Ethics (LIME) Karolinska Institutet Stockholm Sweden; 2 Faculty of Health Sciences Tokyo Metropolitan University Tokyo Japan; 3 Department of Bioinformatics and Telemedicine Jagiellonian University Medical College Kraków Poland; 4 Division for Logistics, Department for Support Services National Home Guard Staff Swedish Armed Forces Headquarter Stockholm Sweden; 5 Department of Research, Education and Development and Innovation Södersjukhuset Stockholm Sweden; 6 Department of Health and Functioning Faculty of Health and Social Sciences Western Norway University of Applied Sciences Bergen Norway

**Keywords:** military trauma, gamification, game elements, serious games, virtual patients, trauma, medical training, medical education, medical assessment, emergency care, first aid, basic life support, trauma care, medics, military

## Abstract

**Background:**

This study explores gamification in the design of virtual patients (VPs) to enhance the training of Swedish military medics in trauma care. The challenges related to prehospital trauma care faced on the battlefield require tailored educational tools that support military medics’ education and training.

**Objective:**

The aim of the study is to investigate how to design VPs with game elements for Swedish military medics to support learning in military trauma care. By understanding the reasoning and perceptions of military medics when interacting with VPs, this study aims to provide insights and recommendations for designing VPs with game elements that are specifically tailored to their needs.

**Methods:**

The study involved 14 Swedish military medics of the Home Guard–National Security Forces participating in a tactical combat care course. Participants interacted with 3 different VP cases designed to simulate military trauma scenarios. Data were collected through think-aloud sessions and semistructured interviews. The data were analyzed using interaction analysis, structured by the unawareness, problem identification, explanation, and alternative strategies or solutions (uPEA) framework, and reflexive thematic analysis to explore participants’ reasoning processes and perceptions and identify possible game elements to inform the VP design.

**Results:**

Mapping the military medics’ reasoning to the uPEA framework revealed that study participants became more creative after making a mistake followed by feedback and after receiving a prompt to make a new decision. The thematic analysis revealed 6 themes: *motivation*, *“keep on trying”*; *agency in interaction with VPs*; *realistic tactical experience*; *confidence*, *“I know that the knowledge I have works”*; *social influence on motivation*; and *personalized learning*. Participants suggested that game elements such as scoring; badges; virtual goods; progress bars; performance tables; content unlocking; hints; challenge; control; imposed choice; narrative; avatars; sensation; randomness; difficulty adapting; competition; leaderboards; social pressure; progression; and renovation can promote engagement, motivation, and support confidence in decision-making.

**Conclusions:**

Gamification in the design of VPs represents a promising approach to military medical training, offering a platform for medics to practice medical and tactical decision-making in a risk-free environment. The insights gained by the study may encourage designing VPs with game elements, as well as including possibly wrong decisions, their consequences, and relevant feedback, that may support military medics’ reflections and decision-making.

## Introduction

### Background

Virtual patients (VPs) have the potential to support the training of competencies required to operate in military settings [1]. VPs are interactive, computer-based simulations of real-life clinical scenarios designed to support health care and medical training, education, or assessment [2]. Gamification, the use of game elements in nongaming contexts [3], is gaining interest in education. Examples of game elements include scoring, avatars, badges, storytelling, and adaptive difficulty levels [4,5]. The incorporation of these elements in education has shown promise in enhancing engagement, motivation, and improving learning outcomes, particularly when these elements promote learning behaviors and attitudes [6]. Gamification appears to be at least as effective as traditional educational methods and often more effective for improving knowledge, skills, and satisfaction in health professions education [7].

Game elements can be combined with other educational methods or technologies. For instance, when gamification is combined with extended reality technologies, such as virtual reality and augmented reality, it offers a powerful approach to modern education by creating immersive and interactive learning environments that make learning more engaging and motivating. The integration of gamification with extended reality can thus transform traditional pedagogical methods, leading to more meaningful and student-centered learning [8]. Applying game elements in the design of VPs for military trauma training has the potential to provide learners with active learning opportunities to prepare for providing medical services under austere conditions [9].

In Sweden, the Home Guard–National Security Forces [10] is a military reserve force of the Swedish Armed Forces that consists mainly of local rapid response units. In addition to personnel who have completed their national service or basic military training, the Home Guard includes a large proportion of various specialists, including military medics.

The Swedish model of military prehospital emergency care has 3 levels, where the first level expects that a wounded soldier on the battlefield shall receive first aid from another soldier or a military medic as soon as possible [11]. The education and training to become a military medic requires essential skills vital to wartime scenarios, where medics are tasked with providing initial basic life support and assisting military nurses or physicians.

Prehospital trauma care on the battlefield differs significantly from that practiced in the civilian sector, particularly in terms of the types and severity of injuries encountered. Military medical personnel face additional challenges in providing care to their wounded teammates under tactical conditions. Unlike natural disasters or civilian incidents such as traffic accidents, where the immediate danger typically subsides and care can be administered in relative safety; battlefield medics might need to deliver care while under hostile fire; frequently dealing with multiple casualties; limited resources; and harsh environmental conditions such as rain, cold, and darkness.

Hemorrhage is the leading cause of preventable death in both military and civilian trauma, making the outcomes of care provided in tactical settings critically important. As gun violence rises in some European countries, injuries more commonly associated with the battlefield, such as gunshot and blast wounds, are increasingly seen in civilian life, representing a growing public health concern [12]. However, the nature of gunshot wounds caused by military ammunition often differs from those typically encountered in civilian settings, primarily due to the higher velocity and a greater kinetic energy of military rounds, which result in more extensive tissue damage [13].

Treatment guidelines developed for the civilian setting do not necessarily translate well to the battlefield. Sometimes, solutions need to be improvised [14]. Courses such as the Tactical Combat Casualty Care (TCCC) initiative are a set of evidence-based, prehospital trauma care guidelines customized for use on the battlefield. These guidelines may be used by battlefield medics, corpsmen, and pararescuemen to acquire trauma management strategies that integrate medical principles with effective small-unit tactics. The curriculum for this training outlines specific objectives, emphasizing the military medics’ need to comprehend the effects of trauma on the human body, administer subsequent treatment, and deliver initial prehospital care. Following the training, military medics are expected to be able to make critical decisions on the battlefield, combining medical care with tactical decision-making [15].

While military medics across different armed forces may have varying duties and organizational structures, their exposure to handling trauma patients is limited. This challenge is further compounded by a lack of research on the learning processes and educational strategies tailored to support the training of military medics [14].

The educational value of VPs can be understood through the experiential learning theory [16], which emphasizes the importance of action and reflection in a safe environment that can tolerate errors [17,18]. VPs may offer a potential solution in preparing military medics by providing a controlled environment where they can practice their skills, make critical decisions, and learn from their mistakes without real-world consequences, ensuring they are better prepared for situations they may face in the field. The risk-free environment offered by VPs has been previously emphasized as their key characteristic in a systematic review of VP descriptions [19]. Through VP simulations, military medics can experience a breadth of scenarios, from the common to the rare and complex, ensuring they are better prepared for situations they may face in the field.

Despite the potential of VP simulations to enhance military medical training, there is a lack of studies focused on incorporating gamification into the design of VPs for military medics. This gap highlights the need to explore how to effectively integrate game elements into VP design to meet the unique demands and learning objectives of military trauma education while increasing engagement.

### Aim

The aim of the study is to investigate how to design VPs with game elements for Swedish military medics to support learning in military trauma care. By understanding the reasoning and perceptions of military medics when interacting with VPs, this study aims to provide insights and recommendations for designing VPs with game elements that are specifically tailored to their needs. The study has two research questions:

1. How do Swedish military medics reason when interacting with the VPs?
2. What are the military medics’ perceptions of VPs?

## Methods

### Study Setting and Participants

A course took place from October 21 to 28, 2023, in a military training area located in Väddö, Sweden, as part of the TCCC training for the Swedish Home Guard. The training aimed to teach Home Guard soldiers basic concepts of TCCC (as part of the TCCC Combat Lifesavers course [TCCC-CLS]) based on the published guidelines [20,21]. This was the first time the TCCC-CLS course took place for the Swedish Home Guard, using the US-standardized TCCC training guidelines and material. The course was attended by 30 participants; 14 (47%) participants were recruited to participate in the study with purposeful sampling. Participants with different professional backgrounds were included to achieve variation in the data [22]. In particular, 11 (37%) participants were soldiers of the Home Guard in Sweden with mixed backgrounds and 3 (10%) participants were course instructors.

### Data Collection

The data collection took place on October 27, 2023, in Väddö, Sweden. A think-aloud method was used while each participant interacted individually with the 3 VP cases, to answer the first research question. Directly after the think-aloud sessions, each participant discussed the cases in semistructured interviews. Two authors (NS and KK) conducted think-aloud and semistructured interviews with the 14 participants.

### Development of the VPs

Three VP cases were developed using the Open Labyrinth open-source system [23]. The content of the VP cases was cocreated with the support of the Home Guard of Sweden in collaboration with the instructors in combat casualty care. The content was designed first in a PowerPoint (Microsoft Corporation) format. To inform the decision points and the content of the VPs, recorded sessions took place with Home Guard members with mixed backgrounds, specifically educators with backgrounds in medicine, nursing, ambulance nursing, combat casualty care, and TCCC-CLS. The 3 cases addressed trauma management caused by gunshot and blast injuries. The cases included key medical and tactical decisions. The medical decisions concerned the use of the <c>ABCDE protocol [21] where the “c” relates to stopping the catastrophic bleeding. Because the medics learn the <c>ABCDE protocol and the military alternative “MARCH” (massive hemorrhage, airway, respirations, circulation, head injury, and hypothermia), the VPs were developed without explicitly referring to the letters of the acronyms, as these can easily be memorized without full understanding of each letter’s meaning. For instance, instead of phrasing a decision alternative as “select A,” the VPs listed the decision as “select to check the airway.”

### Design Principles

The VP cases were developed using design principles identified in the literature and the authors’ own experience. Photos from exercises and educational materials of the Home Guard in Sweden, along with images generated using OpenAI’s DALL-E artificial intelligence system [24], were used. A key design principle was to “emphasize a feeling of presence by keeping the patient visible.” To maximize the sense of presence and not let the interaction become an overly theoretical exercise, the trauma patient was kept visible during every decision the learner made as if the patient was in front of the learner.

We aimed to emphasize the participants’ control and the feeling of being in charge rather than a spectator. The interaction with the VP focused on the user’s next step, enhancing emotional involvement and placing the responsibility for patient care on the learners, requiring them to act quickly and manage the visible trauma. VPs were designed to prompt learners to make decisions as if they were a military medic using first-person language, for example, “I decide to approach the injured soldier.” This approach aimed to immerse the learner in the role and responsibility of a military medic. Incorrect medical decisions, such as failing to prioritize stopping catastrophic bleeding, would result in patients dying from hemorrhage. Suboptimal tactical decisions endangered the user’s character, potentially resulting in injury or death.

The VPs were designed as linear scenarios [25]; if a wrong decision was made, the learner was asked to try again. By clicking “try again,” the learner would be navigated back to the previous key decision node, either medical or tactical, and asked to try again. There was one possible pathway for all VPs, leading to one final case outcome.

A 2-fold scoring system was developed to gamify the VPs, awarding or deducting 10 points for every correct or incorrect medical or tactical decision. Figures S1-S3 in Multimedia Appendix 1 visualize the 3 VPs, with TKD representing tactical key decisions and MKD representing medical key decisions. Table S1 in Multimedia Appendix 1 provides an example of the key decision content of VP1.

The 3 cases were designed with varying levels of difficulty. The first case was the simplest medically, while the third was the most complex. However, the first case involved massive bleeding, making its simplicity not immediately apparent. The scenario provided hints about suspicious objects in the remote area, requiring the user to prioritize tactical safety before assisting the patient. The second case was somewhat more challenging, involving facial bleeding that needed to be controlled in a remote area with an active threat, requiring the medic to approach the patient carefully while taking cover behind stones. In the final case, the patient initially appeared to be in good condition—smiling but slightly confused—yet eventually died due to brain bleeding. In all 3 scenarios, the learner assumed the role of a military medic, tasked with aiding an injured soldier. Each case began with a critical tactical decision, where a wrong choice led to the medic’s death, either from stepping on a cluster bomb or coming under enemy fire. Prioritizing safety was essential in all cases, even though it was not always immediately obvious.

### Piloting the VP Cases

Two researchers, associated with Karolinska Institutet, both with civilian nursing backgrounds and specific experience with medical education, gamification, and civilian emergency and medical education, respectively, were contacted for piloting the VP cases. The 2 researchers conducted the think-aloud sessions and participated in the semistructured interviews. At the last step, they provided feedback as experts, using experience from gamification, emergency medicine, and medical education. After receiving feedback and improving the VPs, the content was validated with the Home Guard combat casualty care course leader.

### Think-Aloud

Think-aloud is a technique where the participant is asked to talk-aloud while solving a problem. Participants are encouraged to verbalize their thoughts as they work through the task. Think-aloud is a method that, in principle, does not lead to much disturbance of the thought process [26]. In our study, participants were prompted to elaborate on their decision-making while playing the cases.

### Interaction Analysis

Interaction analysis was used to analyze the video-recorded think-aloud sessions. Interaction analysis brings focus on the important moments in the learning activity, such as the interaction of military medics with the VPs and their reasoning while making tactical and medical key decisions. This method eliminates the researchers’ bias by grounding assertions on the video-recorded sessions, making them verifiable [27].

The primary objective was to identify recurring patterns of user actions while interacting individually with the VPs. First, NS and KK reviewed the video-recorded think-aloud sessions. We observed that the participants’ comments increased considerably whenever they made a mistake. Therefore, we decided to map the data from the think-aloud sessions to the unawareness, problem identification, explanation and alternative strategies or solutions (uPEA) framework to analyze how they reasoned in relation to becoming aware of problems and dealing with those, particularly after encountering consequences of wrong decisions. Our analysis followed a deductive-inductive approach to qualitative data analysis [28]. The uPEA framework has been previously used to analyze participants’ reflections during post-simulation debriefings [29].

### Semistructured Interviews

Semistructured interviews were conducted to understand how the military medics perceive the VPs and how game elements can inform the VP design. An interview guide with questions was developed by the first author, reviewed by 2 additional authors (AAK and KK), and the questions can be found in Table S2 in Multimedia Appendix 1.

### Thematic Analysis

To analyze the semistructured interviews and identify patterns and themes in the dataset, a reflexive thematic analysis was used following the 6-step approach suggested by Braun and Clarke [30]. The thematic analysis was conducted by the first author (NS) and discussed with AAK and KK in a collaborative and reflexive manner, aiming to achieve richer interpretations of meaning. First, the transcripts were generated using the automatic transcription tool Word (Microsoft Corporation) “transcribe.” NS actively revisited all interviews and recordings and went through the automatically generated transcripts to edit them and generate preliminary notes; then codes were created to encapsulate the meaning of the text. In an iterative manner, themes were actively developed and revisited to form the results and emphasize themes relevant to the research questions.

An additional round of coding was dedicated to associating the participants’ statements to game elements. Using a framework by Toda et al [4] and Maheu-Cadotte et al [5], which we previously used in our research, we performed this mapping organically following a deductive-inductive approach. We actively connected phrases from participants’ discussions to game elements based on our experience in gamification.

### Reflexivity

The reflexive thematic analysis as outlined by Braun and Clarke [30] is a widely recognized method for identifying, analyzing, and reporting patterns (themes) within data. Their approach emphasizes the importance of the researcher’s reflexivity in the analysis process. NS has a background in health informatics and an interest in educational technologies, particular VPs, and gamification. This background has been informing the way that the interview data were interpreted. KK has a background in interaction design and is a senior researcher within the field of medical education, with a particular interest in technology-enhanced learning. AAK has a background in computer science and is experienced as a researcher in topics around technology-enhanced medical education and clinical reasoning.

### Ethical Considerations

Ethics approval was sought from the Swedish Ethical Review Authority (diarienr 2016/1701-31), followed by an amendment in 2020 (diarienr 2020-01660). The Swedish Ethical Review Authority exempted the study from the requirement for ethics approval, as it did not involve handling sensitive personal data, as defined by the Swedish Ethical Review Act.

The video recordings were limited to capturing the laptop screens used, demonstrating participants navigating through the VPs with synchronized voice audio tracks of the participants. No identifiable data about the observed participants were collected. Recordings began only after each participant received oral and written information about the study. Participation could be interrupted at any time without any consequences. All study participants signed a consent form.

## Results

### Overview

A total of 14 military medics participated in the think-aloud and interview sessions. All participants completed the 3 VP cases. The VP cases and the nodes visited by the participants are visualized in Figures S4-S6 in Multimedia Appendix 1. Most visited nodes are visualized with dark gray ranging to light gray and white for less-visited nodes and nodes not visited at all. The number within each node shows the number of clicks per node.

### Swedish Military Medics’ Reasoning When Interacting With the VPs

#### Overview

**Figure 1:**
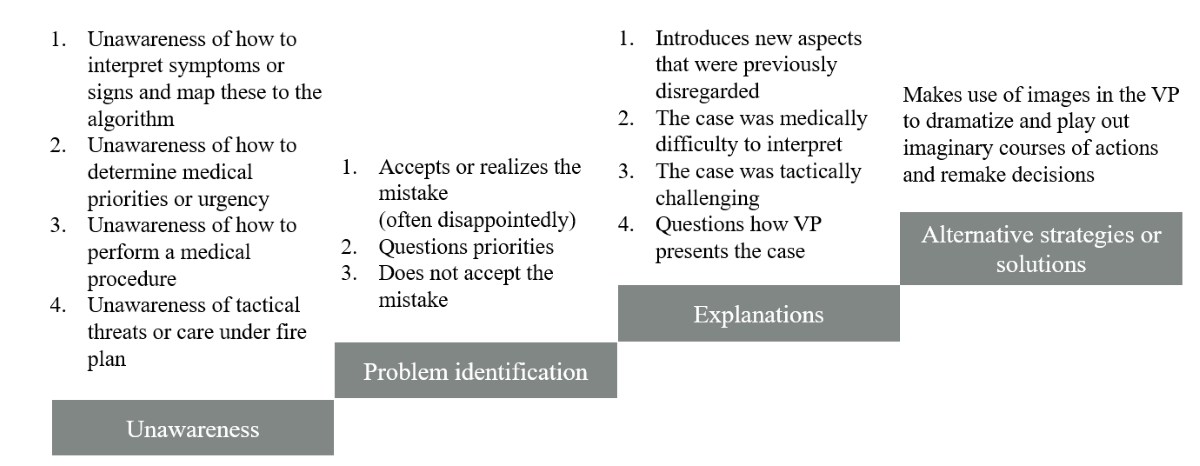
An overview of different kinds of reflections observed during the different phases of the unawareness, problem identification, explanation and alternative strategies or solutions framework. VP: virtual patient.

The uPEA categories were mapped to the participants’ discussions around key decisions as follows: “unawareness” was used to refer to how participants were unaware of a problem that they would subsequently encounter and become aware of. The participants could, for example, comment on the background information, text, and images seemingly unaware of any pending danger or risks just before making a mistake. “Problem” was used to refer to reflections on how the participants realize that they made a suboptimal or incorrect decision. At this point, they discover that the consequences of their decision were not the desired ones. “Explanation” was used to categorize discussions and explanations of why the problem occurred, for instance, to justify a wrong decision made by the participant. The category “alternative strategies or solutions” was used to refer to alternative decisions made after recognizing the problem and considering actions that would avoid similar issues in the future. An overview of different kinds of reflections made by the participants during the different phases is presented in Figure 1.

**Figure 2:**
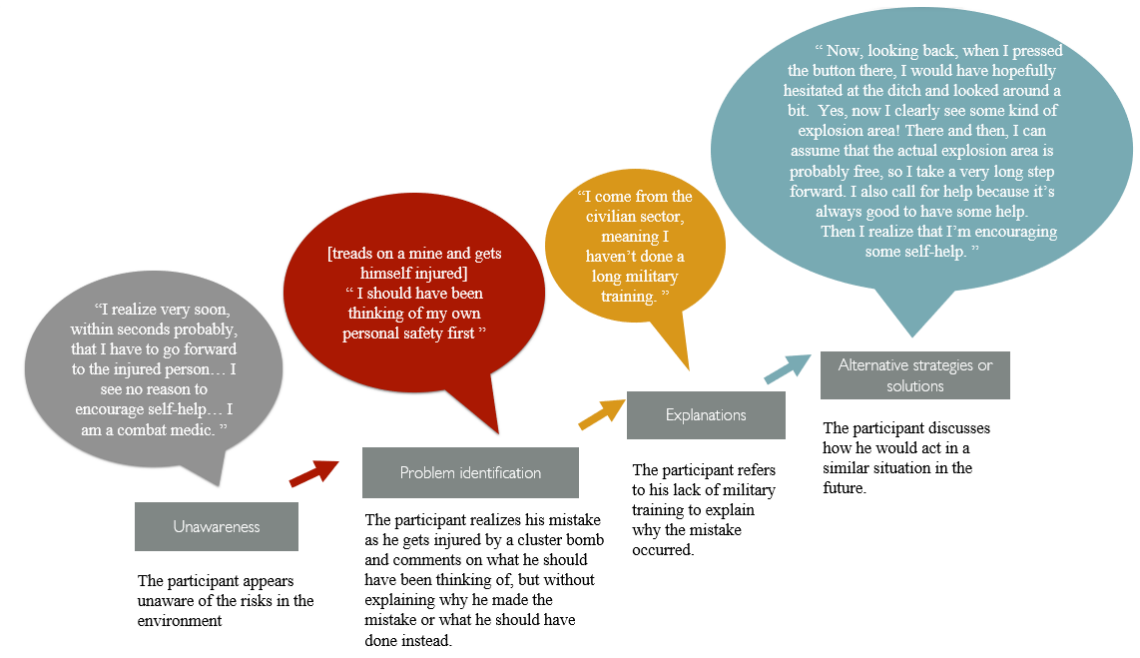
An example of a participant’s reasoning mapped to the unawareness, problem identification, explanation and alternative strategies or solutions framework (participant 10).

An example of a participant’s (participant 10) reasoning, mapped to the uPEA framework, is presented in Figure 2. The example outlines the participant’s reasoning process, starting with his initial unawareness of the risks as he decides to proceed toward the injured patient without hesitation. As he moves forward and steps on a mine, he realizes that he had overlooked the danger, identifying this as the problem (P, which stands for problem identification). He then provides an explanation for his mistake, attributing it to the brevity of his military training (E, which stands for explanation). Finally, he elaborates on how he would act differently in a similar situation in the future (A, which stands for alternative strategies or solutions).

The interaction analysis and the uPEA mapping allowed us to generate the following categories that bring insights about how military medics reason while interacting with the VPs.

#### Unawareness

##### Overview

Participants were often unaware of factors that contributed to the error that they would later make. They seemed not to reflect on the aspects that they would later bring up as important in their explanations. For instance, participants would exclaim that they should hurry to the wounded patient without encouraging self-help because it was part of their role as a combat medic without mentioning the risks involved. Alternatively, looking at an image of a wounded patient concluded that the patient would probably not die, thus apparently unaware of the risk that the patient had latent fatal injuries. In subsequent sections, we have categorized different themes of unawareness.

##### Unawareness of How to Interpret Symptoms or Signs and Map These to the Algorithm

In the first VP case, the term “massive bleeding” was used, and all participants recognized the necessity to prioritize it and stop the bleeding. Thus, everyone made the correct medical decision in the first VP. However, in the second case, several military medics were confused when the term “pulsatile bleeding” was introduced instead of the usual term “catastrophic bleeding” and had difficulty mapping the <c>ABCDE algorithm to the case, even if there were hints of the necessity to prioritize to stop the bleeding. This implies that even if the participants have been trained to the protocol with clear examples of what step to prioritize, they had difficulties applying the same concept to a broader case without a clear hint referring to “catastrophic” bleeding:

There are many things to do, but I need to prioritize cleaning the mouth so that he can breathe. It would be good to stop the bleeding too...But it [the injury] is on the face and jaw...But there is pulsating bleeding in the injured area...It is not catastrophic bleeding. Then one could try...I am between these two [pointing at two options, to stop the bleeding vs to clean the mouth]. I will select this one thought [to clean the mouth].

Participant 5

##### Unawareness of How to Determine Medical Priorities or Urgency

In the second VP case, the patient with trauma on the face caused confusion because of the anatomical location, as the trauma was around the missing jaw. Many participants thought that they should try to free the airway and prioritize breathing instead of stopping the bleeding:

I will try to see whether there are things one can see [in the mouth] so that I can take them out. And then I will try to stop the bleeding, but this is after I check the airways, so this is the order I would follow...

Participant 4

In the third VP case, a picture of a smiling patient appears, who has internal brain bleeding. Although the case is considered medically challenging, several medics understood that there might be a head injury, even if the medical urgency was not always clear, which resulted in the patient’s death:

I think he has a head injury. He is probably not going to die, and he doesn’t seem to have anything wrong with his limbs...

Participant 11

##### Unawareness of How to Perform a Medical Procedure

While dealing with the second VP case, some participants thought that it was practically impossible to stop the bleeding around the mouth area. Therefore, they proceeded with other priorities. While the anatomical positioning of the injury might have influenced their decision-making, the practical perception of how to stop the bleeding and its feasibility was unclear to some of the participants:

If it is pulsatile bleeding, it is not easy to set something [means like a tourniquet] on the face. So, I will select to free the airways.

Participant 8

##### Unawareness of Tactical Threats or Care Under Fire Plan

Many medics were confused in making a decision between “directing the casualty to move to cover and apply self-aid” and “selecting to run to help the patient.” Directing the casualty and encouraging self-aid is one of the priorities of the first phase of the care under fire or threat, according to the TCCC protocol. However, given the design of the VP, which presented a bleeding patient screaming for help, many medics rushed to help the bleeding patient, which resulted in stepping on a cluster bomb and injuring themselves:

...I am safely approaching the injured person on my knees. Securing my weapon, I realize very soon, within seconds, probably, that I have to go forward [to the injured person] because I see that he probably bleeds a lot. I see no reason to encourage self-help because he is actually an injured person, and I am a combat medic.

Participant 10

#### Problem Identification

##### Overview

The VPs provided feedback to the users after each correct or incorrect decision. Sometimes the feedback was just textual, but often the consequences of a decision were quite concrete, so that it was obvious to users that they had made a mistake. If a user was not careful enough and stepped on a mine, such a decision would result in an explosion injuring them, presented in an image. In this section, we describe the reactions and reflections of the participants when it became clear that they had made an incorrect decision. These varied between acceptance, acceptance with a large portion of disappointment, questioning the priorities that should be made, and not accepting the mistake.

##### Accepts or Realizes the Mistake (Often Disappointedly)

Some participants accepted the mistake after it became obvious to them, through experiencing the consequences or through the VP feedback. Often, realizing and accepting the mistake involved disappointment:

...So is it over now? [after stepping on a cluster-bomb, which resulted in an explosion].

Participant 3

…Oh, no! I should have been thinking of my own safety first.

Participant 5

##### Questions Priorities

Reactions to the wrong decision-making also included questioning the priorities presented in the VP. For example, questioning the priorities in the basic management plan for care under fire or threat, where instead of the tactical priority “return fire and take cover,” the participants had to select “direct casualty to move to cover and apply self-aid if able or when tactically feasible, move or drag casualty to cover” because, in the case, there were no enemies presented:

So, isn’t the first priority to kill the enemies?

Participant 1

##### Does Not Accept the Mistake

In some other cases, the participants did not accept the mistake after it became obvious to them:

This was wrong, ok. I need to read the text again. Aha, ok, it states that it is pulsatile bleeding. It is just that I read wrongly. Yes, I will stop the bleeding first. It is just that I read wrongly.

Participant 2

#### Explanations

##### Overview

After having made a mistake, the participants would often stop to reflect on why the mistake took place. Participants offered explanations to why they selected a suboptimal option, such as lack of tactical training, and they sometimes also seemed to rationalize their decisions and attribute mistakes to limitations of the VP scenarios or their interpretations. This process often involved participants justifying their actions by uncovering aspects they had not initially considered.

##### Introduces New Aspects That Were Previously Disregarded

There is no sign of threat in the picture; that’s why I thought of running to help [the patient]. There can be more cluster-bombs on the way, and that’s why I would follow the same pathway as him. But of course, there are risks on the way...

Participant 3

##### The Case Was Medically Difficult to Interpret

It was wrong, yes, but it is a bit difficult to stop the bleeding on the mouth area...That’s how I thought [and skipped it]. When the patient looks like that [pointing at the picture], then I should clear the mouth because otherwise he will not have a free airway. But if it is catastrophic bleeding, then of course I will have to press [stop the bleeding] first...But the picture is limiting the understanding of how serious the catastrophic bleeding is.

Participant 7

##### The Case Was Tactically Challenging

I come from the civilian sector, meaning I haven’t done a long military training.

Participant 5

##### Questions How the VP Presents the Case or Reality

VP limitations that were mentioned to justify wrong decision-making were the (1) picture limitations, (2) a limited number of listed alternatives to choose from, (3) a limited number of options to select at a time, and (4) limited sensory simulation (only sight is involved, and no other senses)

In a real situation, one receives more information. One has a smell, a taste; it is like déjà vu. I think I would perform better if I was there, and I had all these things in front of me, so I don’t need to get all this information [from the VP text]. It would be a long text to give all these details, i.e. how much he is bleeding from the face...But one can’t quantify that either, right?

Participant 5

#### Alternative Strategies or Solutions: Makes Use of Images in the VP to Dramatize and Play Out Imaginary Courses of Actions and Remake Decisions

At the last stage of the reasoning, some of the military medics shifted from justifying their wrong decision-making to reflecting and remaking correct decisions. Not all participants would reflect on alternative solutions and quickly just selected another VP option. However, others engaged in describing scenarios, dramatizing, and playing out imaginary courses of action that they could have chosen instead of making the error. Two examples of participants immersing themselves in the role of a military medic are presented in blockquotes later and in Figure 2. These 2 participants had previously made the wrong decision to quickly approach and help a wounded soldier, without sufficient caution, thereby becoming injured:

I see now that it seems to be in the picture an open territory and that it might be challenging to hide oneself. But there seem to be stones on the ground, and I can imagine that there must be somewhere a bigger stone that I can use to hide myself...If there was a lot of fire going on, I would have approached Sandstedt [the wounded soldier] carefully, ready to exchange fire. If there were enemies around, I would shoot them and prioritize that over Sandstedt, so that we avoid being two dead people.

Participant 1

### Military Medics’ Perceptions of VPs

#### Overview

The thematic analysis resulted in the following themes: *motivation*, *“keep on trying”*; *agency in interaction with VPs*; *realistic tactical experience*; *confidence, “I know that the knowledge I have works”*; *social influence on motivation*; and *personalized learning*. Within each theme, corresponding game elements were developed through the second round of coding and linked to the participants’ discussions. These game elements were inspired by previous studies [4,5] and were further developed and defined based on the authors’ experience. Table 1 presents a summary of the game elements mapped to the themes and defines them within the context of this study.

**Table 1:** Summary of defined game elements and how they map to the developed themes.

Game elements	Definitions	Themes
Scoring	Assigning points for correct actions or decisions and deducting points for incorrect ones to provide feedback and encourage improvement	1, 4, and 6
Badges	Visual symbols of achievement awarded for completing specific tasks or reaching milestones	1
Virtual goods	Digital items earned through performance that can be collected or used within the VP^a^ environment	1
Progress bars	Visual indicators showing the learner’s progress through tasks or learning objectives	1
Performance tables	Detailed overviews of the learner’s performance across multiple tasks or criteria	1
Content unlocking	Access to new content or pathways based on the learner’s performance or decisions	1
Hints	Clues provided to help learners find the correct answers without giving them away directly	1
Challenge	Elements designed to test the learner’s abilities and maintain engagement	1
Avatars	Digital representations of the learners or patients within the VP environment	1 and 2
Control	The player’s ability to influence the game environment and outcomes through their actions	2
Imposed choice	Providing learners with multiple decision options and requiring them to select a specific one to proceed, preventing further progress in the virtual patient scenario until the correct choice is made	2
Narrative	The structured sequence of events and choices that shape the learner’s experience	2
Sensation	The use of sensory stimuli, such as visual and auditory cues, to enhance immersion	3
Randomness	Introducing unpredictable elements into the simulation to create a more realistic and challenging environment	3
Difficulty adaptation	Adjusting the level of difficulty based on the learner’s performance	4 and 6
Competition	Encouraging learners to compete with each other, often through leaderboards or similar mechanisms	5
Leaderboards	Rankings that display top scores to motivate learners through competition	5
Social pressure	Influence from peers or the community that can motivate or demotivate learners	5
Renovation	Opportunities to retry decisions and improve outcomes based on feedback	6
Progression	Tracking and visualizing a learner’s development and achievements over time	6

^a^VP: virtual patient.

#### Theme 1: Motivation, “Keep on Trying”

Motivation was a central theme in the discussion with the participants. While referring to the VPs, participants reflected on features and aspects that could enhance motivation during their interactions. Participants discussed scoring and its potential to increase motivation by encouraging continuous efforts to achieve higher scores. Participants also highlighted badges and virtual goods [5] as elements that could boost motivation and reward good performance. For instance, participants mentioned collecting stars or virtual items, such as virtual food, as incentives for performing well.

Visual progress bars [4] and performance tables [5] were suggested to visualize medical and tactical decision-making progress while interacting with the VPs. For instance, the participants suggested that virtual progress bars with color could depict correct, suboptimal, and wrong decision-making. In addition, participants suggested that branched VPs could unlock content [5] and allow them to interact through avatars [5] with different pathways depending on their decisions, enabling them to explore the consequences of their actions. Content unlocking refers to access to new content or pathways based on the learner’s performance or decisions. This mechanism could motivate participants to perform actions correctly to unlock more content.

Overall, participants appreciated direct feedback after making a wrong decision. However, they suggested that feedback should provide hints [5] of the correct answer rather than giving it away directly to challenge [4] the participants and maintain their interest:

...Progress bars would be good to see where you are, for example, in your way. One can have one progress bar with yellow, green, and red for tactical decisions, and one can have another one yellow, green, and red for medical decisions.

Participant 4

...Say, for example, if you get a certain point, you would then be able to unlock something...You can unlock something for the avatar. It could be anything, it doesn’t have to be anything that really matters, but if I succeed in this [task], then a can of Präriegryta [a stew traditionally served in the military] appears—Congratulations! You have now unlocked Präriegryta! A reward, which may have no impact whatsoever on my ability to solve upcoming tasks. I just think it would be fun, and it would make me wonder, what will happen next? The next thing could be: Here is a package of the Swedish Armed Forces’ skin ointment, it’s silly, but I think the human brain is wired to seek these kinds of rewards, and they don’t have to have any real value...

Participant 5

#### Theme 2: Agency in Interaction With VPs

Agency in interaction with the VPs was discussed in terms of how participants navigate and interact with the VP environment. Participants highlighted the importance of having a sense of control over their actions and decisions within the VP environment. They mentioned that, in certain cases, they felt limited in their decision-making and movement within the environment.

Participants suggested that they would appreciate more freedom in decision-making and how they could move around in the environment. However, answering with just free-text answers was perceived as more challenging; participants suggested that such an option should be combined with listed decision alternatives, known as imposed choice [4]. Imposed choice refers to decisions made from a set of given options, which can help guide the learning process.

The participants also discussed the importance of narrative [4], which is the structured sequence of events and choices that shape the learner’s experience. They believed that being able to navigate within the VP by making choices listed in the VPs, combined with an interactive free-text menu, would give them a feeling of freedom and help unfold the scenario.

In addition, the participants mentioned the importance of the agency provided by avatars. They believed that avatars could enhance the feeling of control over the environment, which could influence motivation positively:

Yes, like it’s “high alert,” okay? But there is always much more information available. How do we assess the threat level right now? Do we have an external barrier in the form of surveillance? Have we set up an alarm system? Is the terrain mined, or can they [the enemy] just walk in? I need to know that! Because I will behave differently based on this information...So, yes, the medical and tactical [scoring], it’s the tactical I am talking about; that’s where it’s very difficult if I can’t make entirely tactical decisions because the information isn’t there. Or the options I would have liked are not there...

Participant 1

When it comes to graphics, I don’t think it is so important in a way, I think that I value the autonomy of the player the most. For instance, I would like to be able to create something by myself if I can give a name to my character or give it my name.

Participant 5

[The interactive menu] gives you a bit more freedom if there is something else you would like to do, but the option is not there.

Participant 9

#### Theme 3: Realistic Tactical Experience

The significance of delivering authentic, realistic experiences through VPs was discussed by the study participants. They highlighted the importance of the tactical environment, which may influence their decision-making. This theme is closely related to theme 2 and how immersion in the virtual environment representation can support the users’ sense of agency. Participants frequently mentioned the visual detection of potential threats or enemies within the VP simulation as an example of realism. Multimedia elements, such as sound, pictures, and video, can enhance the feeling of presence and improve the perception of the virtual environment. Sensation [4], which involves visual and auditory stimuli, was highlighted as crucial for improving the experience. This includes virtual worlds, virtual reality, or augmented reality to engage the senses.

Participants noted that the tactical environment is constantly changing and unpredictable, often intersecting with medical decisions. This interplay makes military medical professionals distinct from civilian ones. Therefore, participants implied that the characteristics of the tactical environment should be highly visible and emphasized in the virtual environment. They suggested using multimedia elements to enhance realism and aid in understanding the tactical environment. Randomness as a game element [4] refers to the unpredictable elements introduced into the simulation, which can create a more realistic and challenging environment. This can simulate the unpredictability of real-life scenarios, making the experience more immersive and engaging. Realistic cases were perceived to enhance engagement and influence the sense of agency in the VP interaction:

I know what I would do, and I know what the game [VPs] would like me to do. The more the war environment and the situation is clarified, the tighter these two become. For instance [in the last case], I didn’t know if they were still shooting, and that’s why I decided to run into the building. If I knew, if there were people screaming, if I knew something was happening...Now my intuition told me that the shooting had stopped, so I wanted to get into the building...It is important with sound; to be able to listen the sounds of a war, someone can hear things before they come.

Participant 3

One gets already a lot of information from the picture...[referring to the second VP]. Someone can embed videos for 5-10 seconds so one gets a better idea of the patient.

Participant 7

#### Theme 4: Confidence, “I Know That the Knowledge I Have Works”

Participants felt that high scoring could increase their confidence, encourage them to continue interacting with cases, and thus enhance learning. They reflected that their previous combat casualty care training was applicable when managing the VPs and that this was reflected in their scores. Alternatively, participants commented that they needed more practice if they did not achieve the highest scores.

Adjusting the difficulty of presented educational content based on the learners’ performance scores is known as difficulty adaptation [5] of the designed cases. Participants suggested that if the tasks are too challenging, they may become discouraged by low scores. Conversely, very easy cases might not challenge learners enough. Participants thought the cases were relevant to the course content, quizzing knowledge they had received, with the first and second cases being medically easier than the third one. Participants suggested that feeling unable to solve cases due to high difficulty levels could negatively influence their motivation:

I learned [from the VPs] that the knowledge I got from the course works [after looking at his final score].

Participant 5

Yes, it’s good [the scoring]! And it is also good to get a patient that isn’t possible to save. It’s good to know...that you don’t [always] have a chance...that you are done. But the percentage of such cases cannot be too high, because then you will lose motivation...It’s the same thing when you talk about game development: if you have too much, too difficult, then people quit. They will lose interest; you have to have some “wins” too.

Participant 4

#### Theme 5: Social Influence on Motivation

Participants mentioned that scoring could be fun and enjoyable, particularly that higher scores increased their enjoyment of interacting with the VPs. They believed that social elements, such as group competition, could be motivating. Competition [4] involves encouraging learners to compete with each other. Leaderboards [5] are rankings that display the top scores of participants, motivating learners through visible performance comparisons. Participants suggested that displaying the top 3 players’ scores to everyone could act as a goal for others to achieve. However, they also thought that low scores should not be visible, as this could embarrass individuals and cause demotivation. Social pressure [4] refers to the influence from peers or the community that can either motivate or demotivate learners. In this context, participants mentioned that visible scores and rankings could exert social pressure, influencing their motivation positively or negatively. While high scores and top rankings can be motivating, low scores can create negative feelings and reduce motivation:

I don’t think that one should have everyone’s score in a list, but maybe one could have a list with the ones having the highest score, let’s say Gustafsson, that has 77 points. Then I want to beat Gustafsson and get a higher score and I will keep on trying. But then on the other hand, the one who is lower on the list, if I had 26 points for example out of 100, then I wouldn’t like it to be visible. Then everyone would see that I have difficulties [with the game] and then I would lose motivation...I think that it would be good if the list just showed the top 3 or 5.

Participant 5

#### Theme 6: Personalized Learning

Participants highlighted the importance of personalized learning, where feedback and progression are tailored to the individual’s performance. Scoring can provide personalized feedback on the number of correct decisions made during interactions with VPs. This feedback helps learners understand their current knowledge level and identify areas needing more practice.

Progression [4] refers to tracking and visualizing a learner’s development and achievements over time. Participants suggested that users could log in and interact with VPs, with the system tracking their progression. This tracking allows users to see their current knowledge level and identify areas needing more practice. Participants also suggested receiving personalized material to practice based on previous performance. This feature aligns with the game elements of scoring and progression, where specific feedback on progress influences participants’ confidence levels and personalized learning paths.

Furthermore, the system could enable users to repeat specific decisions after receiving negative feedback, promoting mastery in learning by allowing users to retry and improve their scores. Renovation [4,31] allows users to face the consequences of their actions and try again once their previous actions have failed. The participants perceived positively the fact that they could have a second chance to make a new decision, after failing with the first one, because it enabled them to realize wrong decisions and improve them.

Finally, participants suggested that they would be willing to practice decision-making with a library of VPs that offer a mix of varying levels of difficulty, allowing for continuous and adaptive learning experiences:

I learned [from the VPs] that I need to practice more...One can complete the VP and get some form of assessment about the performance and then receive tasks to do.

Participant 1

The scoring could be a way to see how well you are doing and someone could perhaps log in and see next time maybe that there are new things and tasks to do. The scoring can provide information about whether one has improved or is at the same level, so that I think it can be a good thing and that one might get a personal login or something so that one can follow the statistics on.

Participant 9

It is very good that one is given the option to try things again after one gets to know what the problem is. Because sometimes one has been making the wrong decision and one continues in the same direction [without noticing the mistake]. Here one knows, ok this is wrong, I am going to try again and make another decision…. I think it is good to get feedback immediately after making a mistake, and then provide them again with different decision alternatives. But it should not give away the right answer, like stopping the bleeding, directly, because then it becomes too easy.

Participant 11

## Discussion

### Principal Findings

This study investigated how to design VPs with game elements for Swedish military medics. The interaction analysis of the think-aloud sessions, structured with the uPEA framework, and the thematic analysis of interviews provided insights about the participants’ interactions and design preferences. The insights gained may encourage designing VPs with game elements, as well as integrating errors that may support military medics’ reflections and decision-making.

Overall, the VPs were positively received and effectively highlighted problems, leading to reflection and discussion. In the first VP case, the medics were confused; they lacked awareness of possible threats posed by the tactical environment and injured themselves by rushing to help patients. A shift was noted where most medics became tactically more aware, resulting in improved tactical decisions in the second and third VP cases. It is likely that the medics realized the consequences of their wrong tactical decisions in the first VP and applied this experience in the second and third VP. This observation shows the potential of VPs as an educational aid to support learning of situational awareness. This pattern was not observed in medical decision-making due to the varied medical challenges in each case, preventing the transfer of insights from one VP to another.

While analyzing how the participants explained their mistakes, we observed that some participants showed reluctance to accept their mistakes, attributing errors to the limitations of the VPs, such as the absence of 3D pictures and audio. They emphasized the importance of authentic and realistic experiences, particularly in detecting potential threats. For instance, being able to visually detect potential threats and enemies in the VPs was frequently mentioned as an example of “realism.” While the 2D pictures and lack of sound do limit the presentation of the environment, this expectation may diverge from the reality of actual military operations, where threats often may remain concealed or unexpected. This raises the question about the role of realism in educational simulations and how fidelity to real-world conditions is realized in simulations [32].

Realism, although highlighted as important by the participants, is not the overall goal of simulation. Using various techniques that enable reflection to support decision-making may be more important. A study by Massoth et al [33] observed that high-fidelity simulation led to equal or even worse performance as compared with low-fidelity simulation. A previous systematic review noted that “presenting realistic patient scenarios with a great degree of freedom cannot be an excuse for neglecting guidance in relation to learning objectives” [17]. In some cases, “departing from realism” can enhance the quality and effectiveness of training by allowing participants to have a second chance, repeat scenarios after debriefings, or slow down the deterioration of a patient to provide time for reflection [34].

Although feedback and prompts for corrective actions may reduce the “realism” of field conditions—where consequences of wrong decision-making may not come with a possibility of correction—it was perceived positively by the participants. Feedback helped them recognize poor decision-making and personalized the learning experience. Unlike dialogue-oriented VPs, which primarily simulate clinical encounters through scripted interactions, our study’s VPs enabled reflection by allowing participants to analyze their decisions and learn from their mistakes.

Purposefully selected multimedia elements, such as pictures, audio, and videos, can enhance the perceived realism and participants’ understanding of the tactical environment. In our study, we used the generative AI application DALL-E [24] to generate realistic patient images in various simulated contexts. Such educational use of the new generation of artificial intelligence tools in VP design has been suggested also elsewhere [35] and seems to be a viable solution to increase realism, situation awareness of the simulation, and learner emotional engagement. Emphasizing the characteristics of the military environment in simulations was perceived as important in our study, as it allows participants to practice dealing with consequences that might be challenging to face in a live simulation.

Some of the participants mentioned feeling constrained while navigating in the VPs because of the limited number of listed decision alternatives they had to choose from. An interactive menu, where one can type in keywords, or a complementary free-text box could enable participants’ wish for agency in decision-making, combined with several listed decision alternatives. In addition, participants mentioned that they value the agency in the interactions with the VPs, for instance, to be able to navigate freely in a virtual environment. Avatars were discussed as a positive feature to enhance motivation and the feeling of controlling the environment.

VPs effectively highlighted errors by showing the negative consequences of suboptimal choices, scoring performance, and providing textual feedback after decisions. What initially seemed like a limitation—such as the lack of sound and videos, which might lead to an incomplete understanding of the tactical environment—actually promoted participants’ imagination and creativity. Many participants progressed from recognizing errors to reflecting on explanations and considering alternative solutions. Similarly, a previous study found that facilitators helped learners shift from focusing on explanations to generating alternative solutions by dramatizing scenarios [26].

In our study, the think-aloud sessions supported this reflection. To more effectively raise users’ levels of reflection, VPs could be designed to prompt learners to consider why a mistake was made and think about next steps before presenting optional paths. Reflective practice and feedback, crucial for developing expertise, have been emphasized in several studies and align well with our findings [36,37]. VPs allow users to make mistakes and receive feedback, providing valuable opportunities for reflection on decision-making. Studies suggest that feedback in VPs can positively influence learning outcomes [17,38].

The participants also argued for various game elements that they believed would enhance the learning experience by making it more enjoyable, increasing their confidence, and providing reassurance that they were making the right decisions. These elements included scoring, badges, virtual goods, progress bars, performance tables, content unlocking, hints, challenge, control, imposed choice, narrative, avatars, sensation, randomness, difficulty adaptation, competition, leaderboards, social pressure, progression, and renovation.

Feedback was suggested to be realized in the way of providing hints for the correct answer and not giving away the right answer directly. Scoring and feedback can add a personalized dimension to learning; participants suggested that they would like to have access to several short cases to be able to log in to the VP platform and receive challenges in the form of new cases to solve. Personalized feedback would allow them to train competencies based on their performance, while it would allow them to monitor their progress and understand which competencies they need to train and improve. Visual progress bars depicting progression and achievements in medical and tactical decision-making were suggested as an alternative to visualize scoring by the participant.

Scoring of the users’ performance was viewed as an element that can motivate users to keep on trying to improve. Although all participants agreed that scoring is a useful feature, many of the participants had not noticed the scores in the VP cases of this study as the scores were not placed in a prominent place on the screen. Scoring has been previously used in studies in various forms, to inform the participants about their progress. In a previous study, scoring was presented in the form of a trauma score [39], tracking the management of the trauma patient. Achatz et al [40] used a similar concept, presenting scores in the form of “health points” to depict how well the patient responded to the decision-making and events taking place in the game. Participants suggested that obtaining good scores makes the learning experience enjoyable and adds confidence, as it indicates that the knowledge they acquired during their training is useful. Similarly, previous studies have demonstrated that scoring-based game mechanics effectively motivated players and enhanced their learning engagement in a Swedish emergency department game [41].

According to the study’s participants, displaying the third to fifth top scores to everyone can motivate learners to compete with each other, reflecting the concept of leaderboards. However, participants also noted that displaying less successful performance in the form of low scores could undermine the learners’ reputation and lead to negative feelings. Social aspects and gaming preferences may vary across different cultures and professions. Some participants suggested integrating competition into VPs to increase motivation. However, other studies have reported that stakeholders in emergency medicine believe competition is not aligned with the culture of health care professionals [41]. Future studies should therefore carefully consider the target end users when designing and implementing competition as a game element.

Participants suggested that future efforts could focus on developing a VP repository with short cases and supporting the creation of VPs tailored to different levels, roles, and experiences of Swedish Home Guard members. Such VP repositories already exist for general medicine [42], and the idea could be transferred to military medicine education. This approach could further explore the potential of accelerating the design of VPs. In addition, future research could examine whether the design principles and game elements are applicable to other military medicine professionals and across different cultural and operational contexts.

According to the literature, serious games may improve performance in life-saving interventions on the battlefield [43,44]. Future research should investigate how gamification and specific game elements can enhance learning outcomes and the long-term retention of skills and knowledge acquired through gamified VP interactions.

We acknowledge that the context and culture of Swedish military medics may not fully generalize to other military or medical training environments. Moreover, the number of participants was limited (14) and therefore caution should be taken when considering how findings may transfer to other contexts. However, it is important to note that this study is qualitative in nature, where the depth and richness of the data are prioritized over the sample size.

While the uPEA framework might suggest that mistakes were the primary focus of the interaction analysis, we used it to understand how participants became more creative and reconsidered decisions when reflecting on mistakes. These insights could inform future VP designs.

The reflexive thematic analysis followed the 6-step approach suggested by Braun and Clarke [30]. While this is an established and flexible approach, it is highly interpretive, encouraging the experiences of the researchers to influence and inform the themes. Although this subjectivity can affect the consistency and replicability of the findings, it also serves as a strength in the context of this study. Our experience and familiarity with gamification strategies allowed coding game elements and informing the developed themes within the semistructured interviews. This capability was crucial, given that the participants were not well familiar with gamification terminology and thus unable to explicitly name these elements themselves. Through the reflexive thematic analysis, our study draws out implicit references to game elements and interprets discussions around participant experiences that are aligned with gamification concepts. This approach leverages the inherent subjectivity of our analysis to produce deeper insights that might otherwise be overlooked by the use of other methodologies.

### Conclusions

Gamification has the potential to enhance VP design and offer flexible learning opportunities to support the education and training of Swedish military medics. The uPEA framework provides a useful lens for understanding decision-making during interactions with VPs, helping to inform how mistakes followed by feedback in VPs can promote reflection and creativity.

Game elements may effectively leverage the potential of VPs by fostering motivation, enabling personalized learning, supporting agency, and enhancing the confidence of military medics. Emphasis may be placed on representing austere aspects and consequences of the tactical environment. Incorporating opportunities to make errors followed by direct, constructive feedback mechanisms in the VPs may further enhance learning outcomes, encouraging critical reflection and continuous improvement.

## Appendix

Multimedia Appendix 1 Visualization of virtual patient (VP) VP 1, VP 2 and VP 3 structure (Figures S1-S3); visualization of learners’ interaction with decision nodes in VP 1, V2, and VP3 (Figures S4-S6); tactical key decision in VP 1 and decision alternatives (Table S1), and semistructured interviews guide (Table S2).

## Abbreviations

MARCH: massive hemorrhage, airway, respirations, circulation, head injury, and hypothermia
TCCC: Tactical Combat Casualty Care
TCCC-CLS: Tactical Combat Casualty Care Combat Lifesavers course
uPEA: unawareness, problem identification, explanation, and alternative strategies or solutions
VP: virtual patient
