# Supplementary material for: Gamification in the Design of Virtual Patients for Swedish Military Medics to Support Trauma Training: Interaction Analysis and Semistructured Interview Study
Source: JMIR Serious Games. 2024 Oct 22;12:e63390. doi: 10.2196/63390 (PMC11538879; doi:10.2196/63390)
Supplement: Multimedia Appendix 1 [file games_v12i1e63390_app1.pdf]

Figure S1

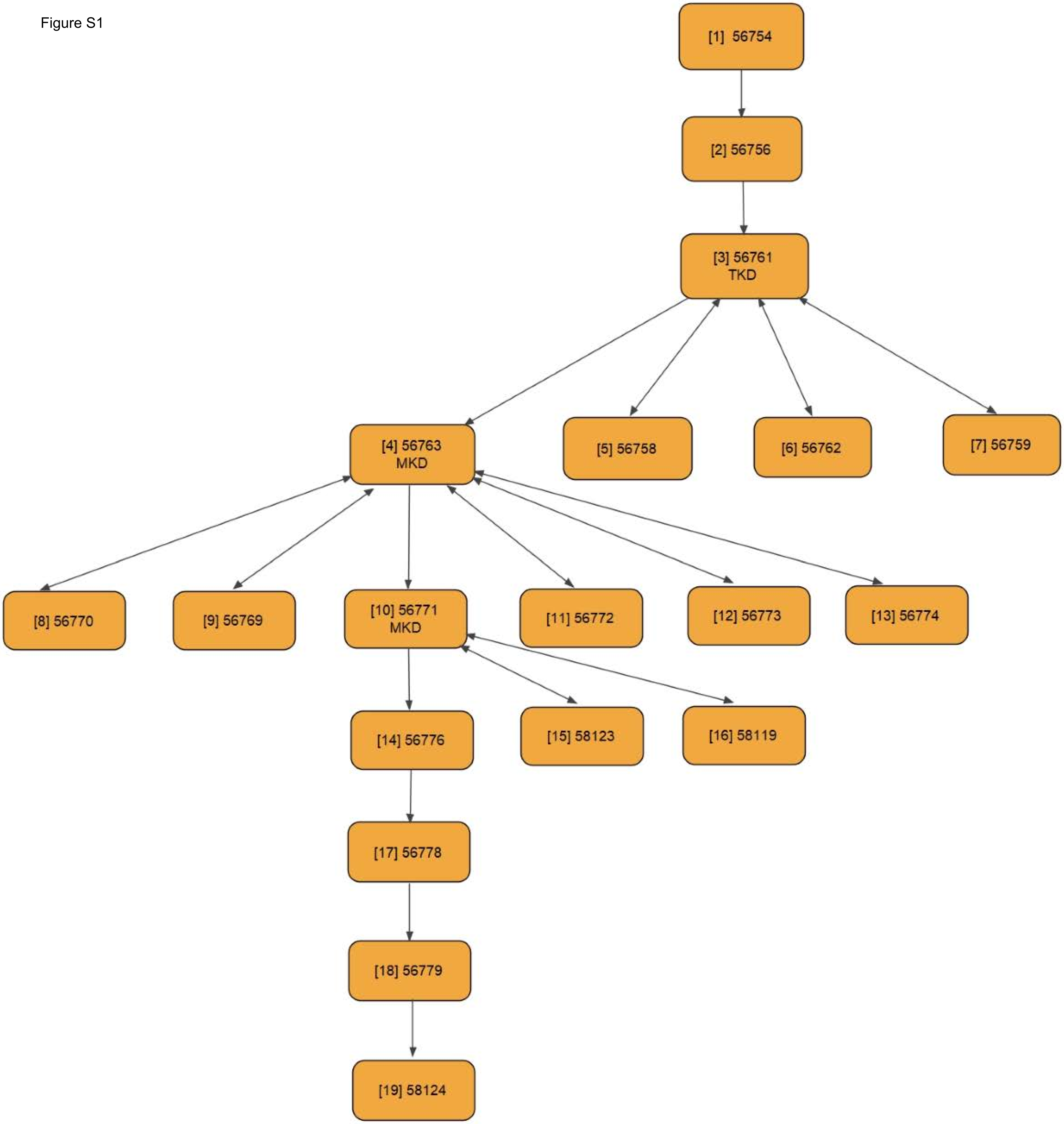

[1] 56754
[2] 56756
[3] 56761
TKD
[4] 56763
MKD
[5] 56758
[6] 56762
[7] 56759
[8] 56770
[9] 56769
[10] 56771
MKD
[11] 56772
[12] 56773
[13] 56774
[14] 56776
[15] 58123
[16] 58119
[17] 56778
[18] 56779
[19] 58124

Figure S2

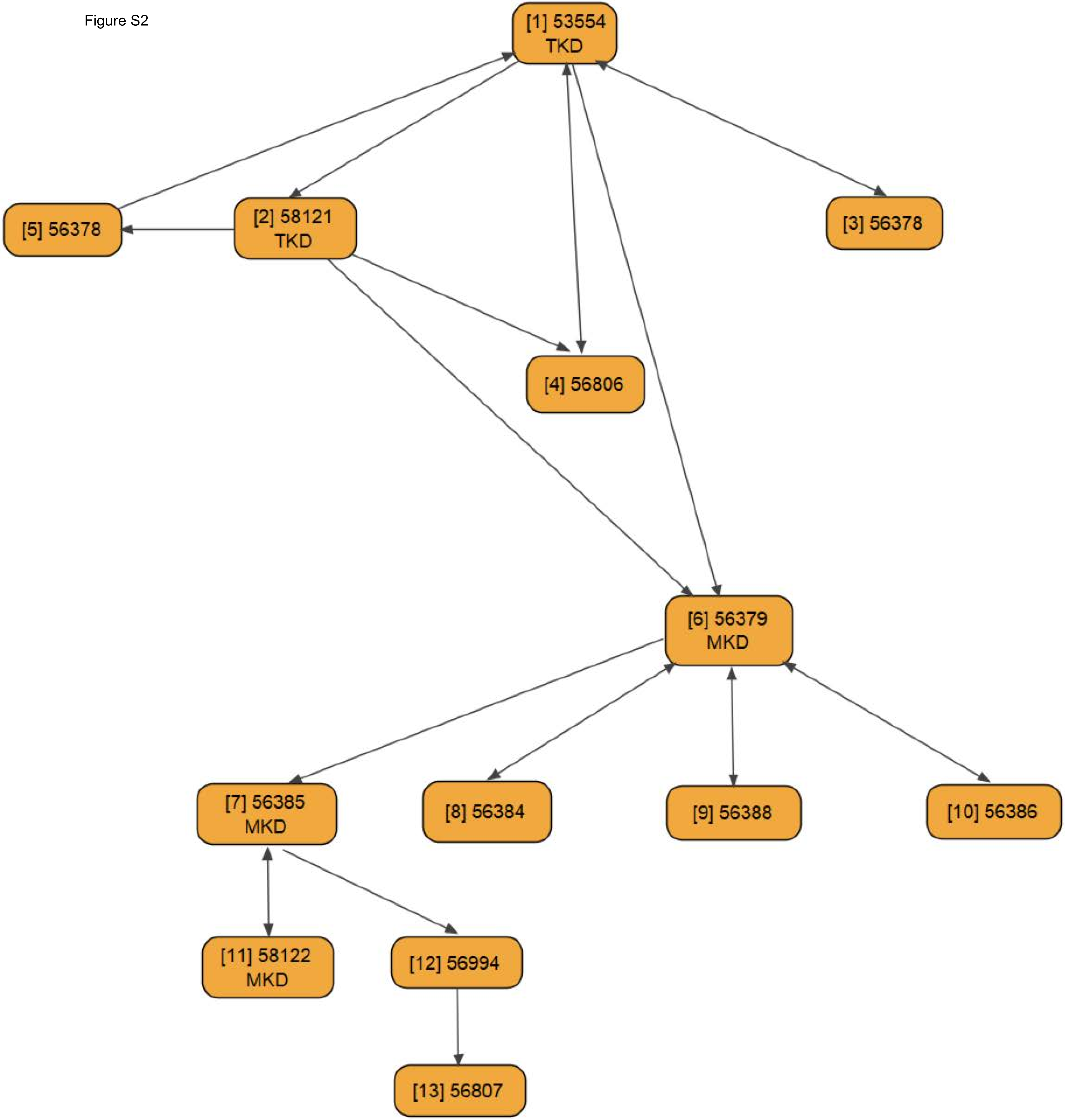
[1] 53554
TKD
[5] 56378
[2] 58121
TKD
[3] 56378
[4] 56806
[6] 56379
MKD
[7] 56385
MKD
[8] 56384
[9] 56388
[10] 56386
[11] 58122
MKD
[12] 56994
[13] 56807

Figure S3

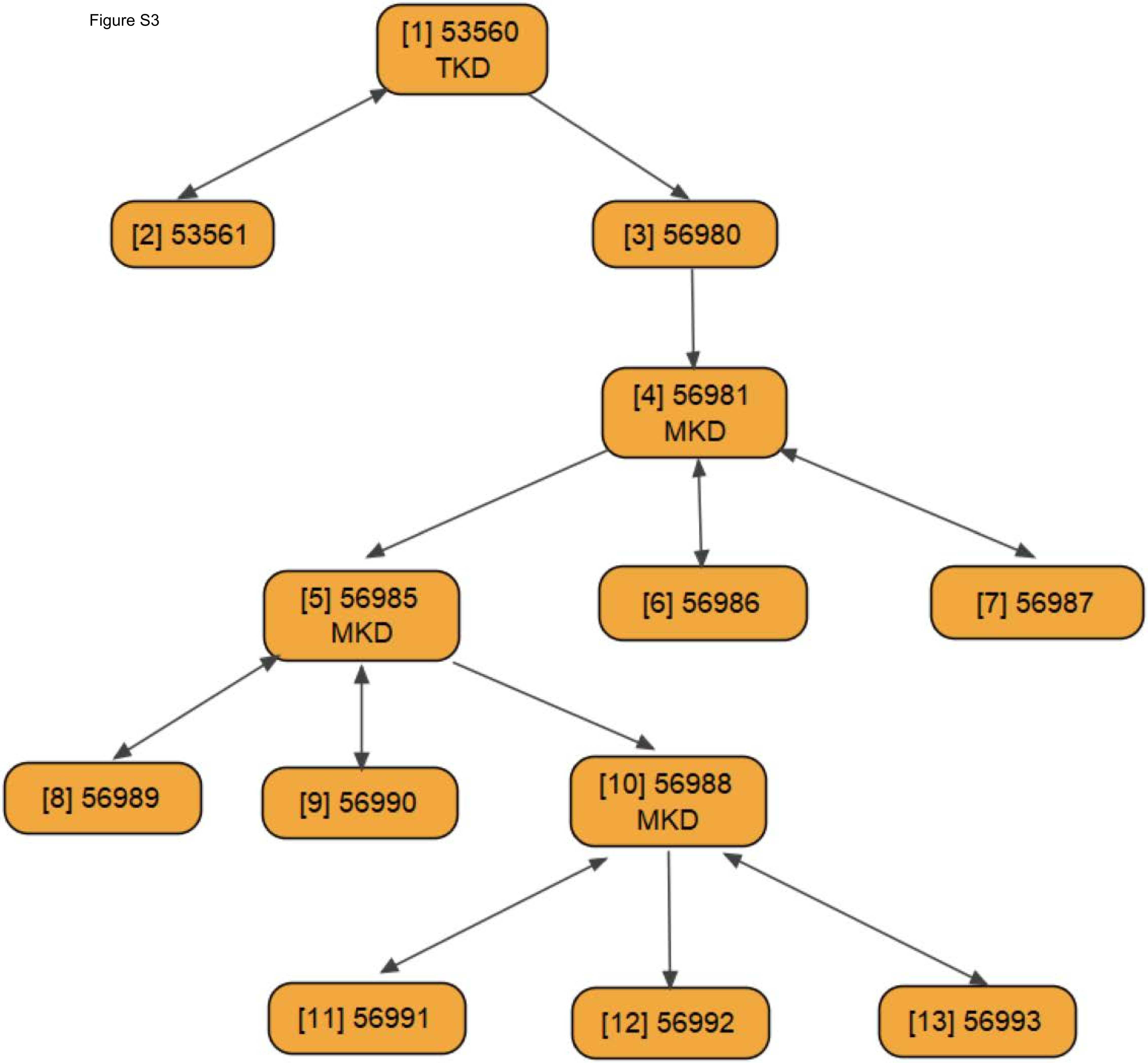
[1] 53560
TKD
[2] 53561
[3] 56980
[4] 56981
MKD
[5] 56985
MKD
[6] 56986
[7] 56987
[8] 56989
[9] 56990
[10] 56988
MKD
[11] 56991
[12] 56992
[13] 56993

Figure 4

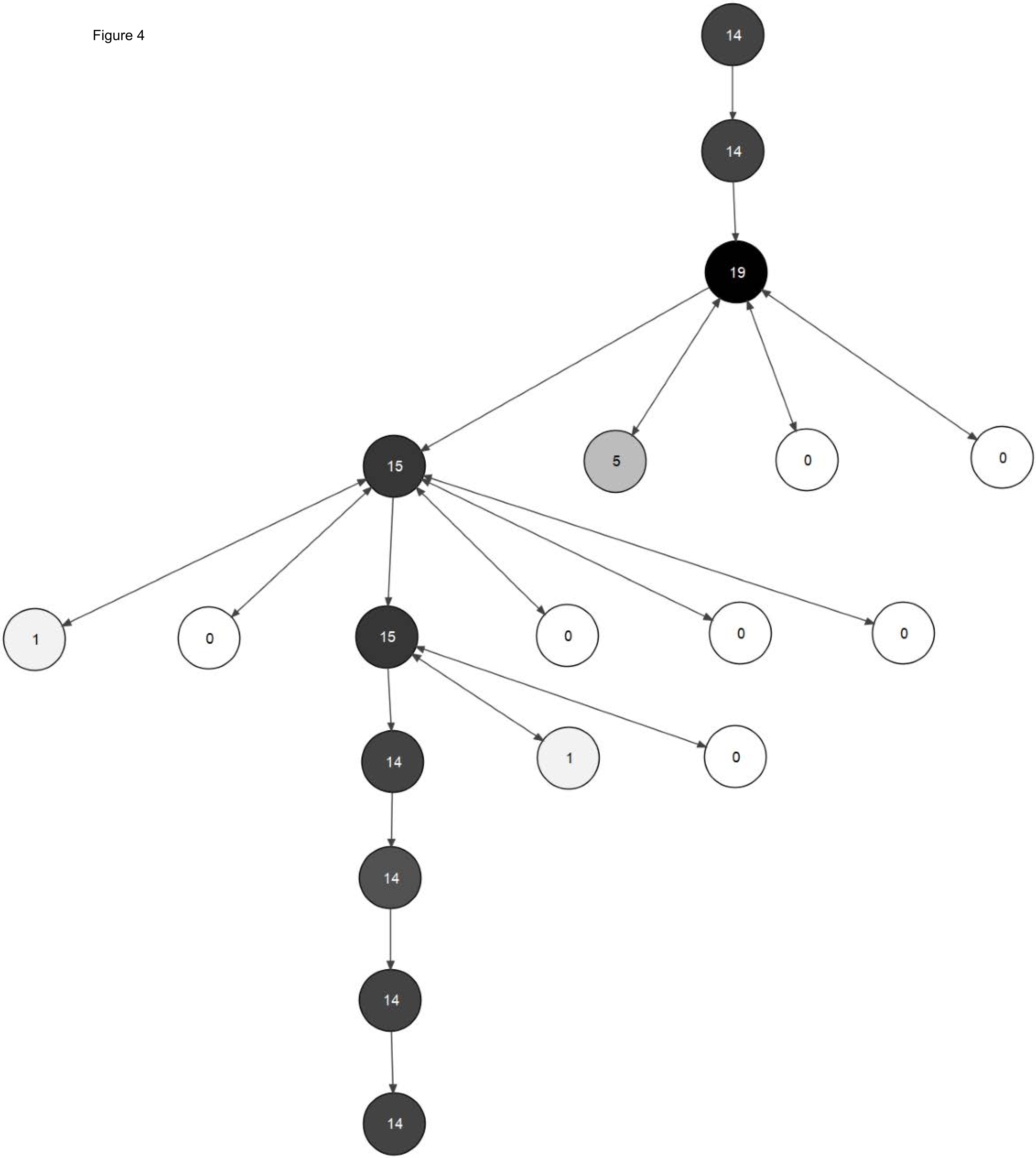

Figure S5

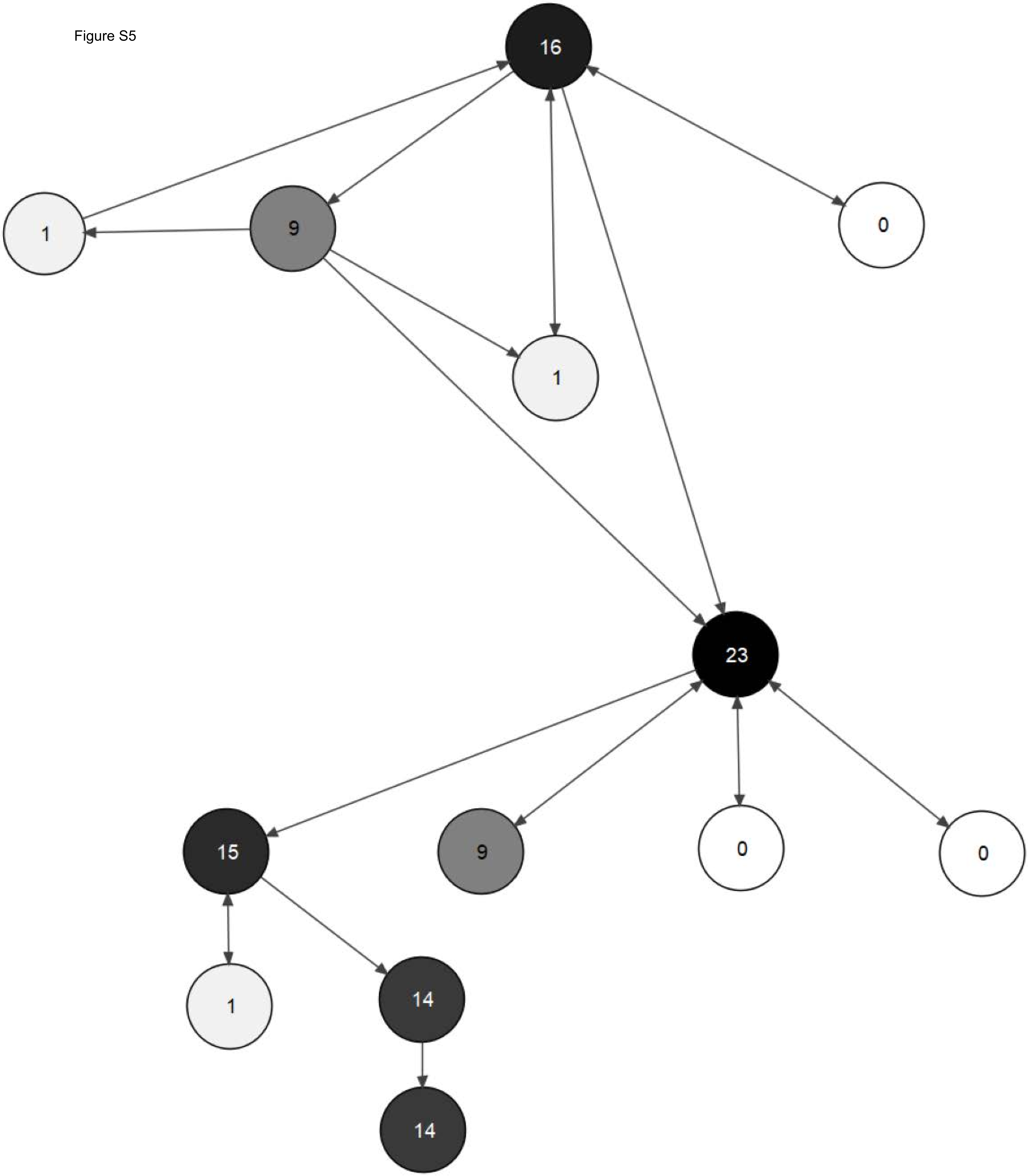

Figure S6

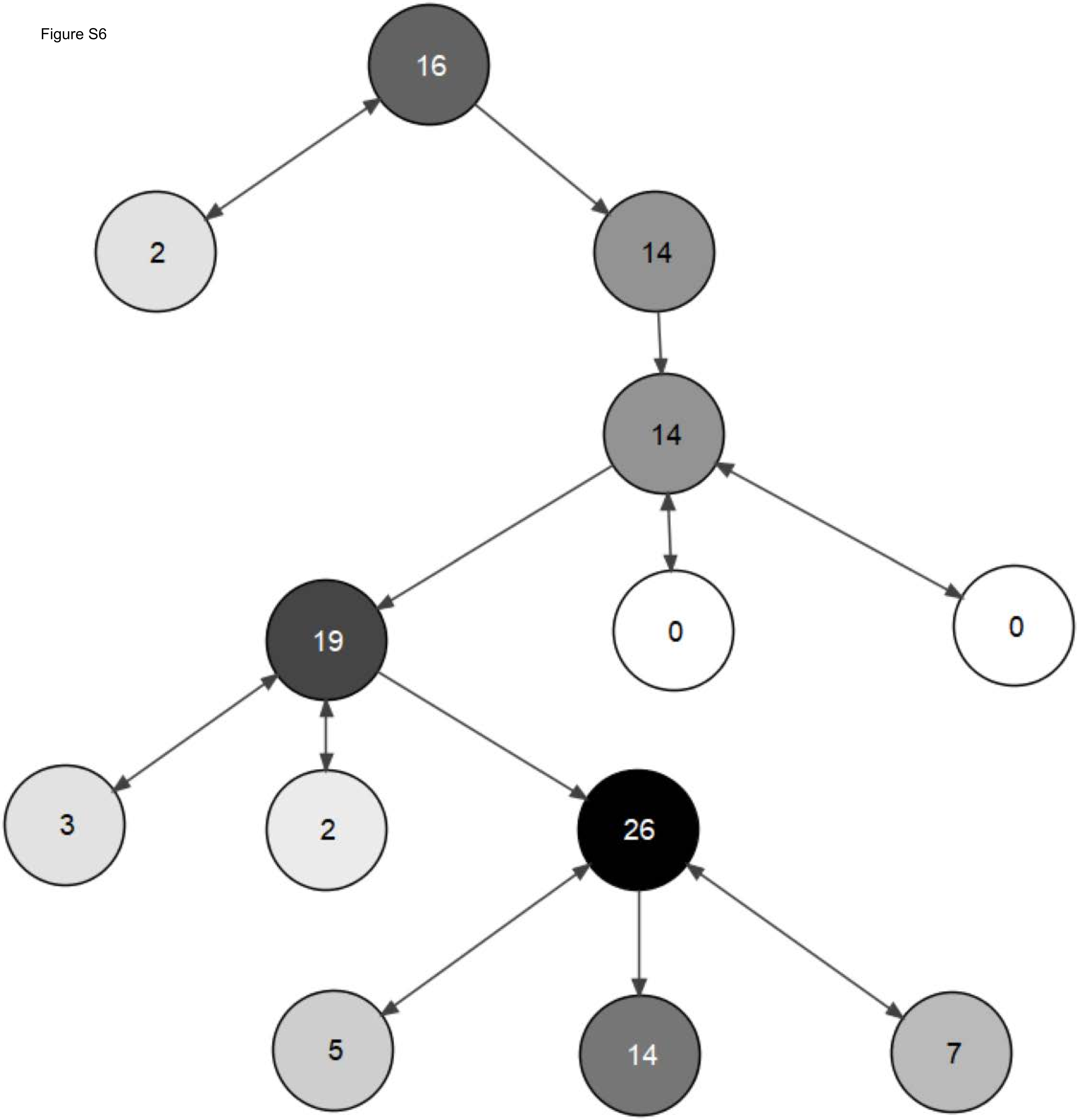

Table S1: Tactical key decision in VP1 and decision alternatives

Screen card	Valence	Content
Tactical Key Decision	Link to Tactical decision alternative 1, 2, 3 and 4	The commander lies bloody in the ditch.<br><br>You are the first person on the scene, and you see that the commander is bleeding profusely. You must act fast! What do you do first?
Tactical decision alternative 1	Correct decision	Encourage till self aid
Tactical decision alternative 2	Wrong	I rush to help the commander
Tactical decision alternative 3	Wrong	I will call Sundberg who is standing at the warehouse, he is capable at proving
Tactical decision alternative 4	Wrong	I am shouting that I need help
Result of tactical decision alternative 1	Proceed with the case	Good, you call for the commander to apply a tourniquet and then lie down in the prone position. Approach the casualty carefully. It can be good to search the surroundings for possible cluster bombs or other dangers before running to the injured person. Therefore, make your way to the injured person through the tall grass, or stay on hard ground.

Result of tactical decision alternative 2	Go to the previous step (tactical key decision) and re-make a new decision	You don't quite remember what happened but now realize that you must have stepped on a cluster bomb yourself. You are also lying in the ditch and cannot move your legs.<br><br>Help yourself first by e.g. put a tourniquet. Lie down on your stomach. Take cover if possible. Encourage the company manager to help himself.

Table S2: Semi-structured interviews guide

Do you have any previous experience with virtual patient cases? (If yes, then what?)
What do you think you learned using the virtual patient cases? Was anything particularly eye-opening?
What do you think about the difficulty level of the virtual patient cases?
What do you think about the level of medical and tactical decision options in the virtual patient cases?
Were the decision-making options realistic?
Would you use the virtual patients in your spare time to practice your skills? Why or why not?
Would you like to try the virtual patients again? What did you think of the stories in the scenarios?
What do you think about the point system in the virtual patient cases? Does it affect your motivation?
Overall, what do you think of the virtual patient cases? Do you have any other reflections and comments you would like to share with us?
